# Four New Glycosides from the Fruit of *Xanthium sibiricum* Patr.

**DOI:** 10.3390/molecules181012464

**Published:** 2013-10-10

**Authors:** Hai Jiang, Liu Yang, Chang Liu, Hui Hou, Qiuhong Wang, Zhibin Wang, Bingyou Yang, Haixue Kuang

**Affiliations:** Key Laboratory of Chinese Materia Medica, Ministry of Education, Heilongjiang University of Chinese Medicine, Harbin 150040, China

**Keywords:** *Xanthium sibiricum* Patr., fruits, glycosides

## Abstract

Four new glycosides, namely 3β-norpinan-2-one 3-*O*-β-d-apiofuranosyl-(1→6)-β-d-glucopyranoside (**1**), (6*Z*)-3-hydroxymethyl-7-methylocta-1,6-dien-3-ol 8-*O*-β-d-glucopyranoside (**2**), (6*E*)-3-hydroxymethyl-7-methylocta-1,6-dien-3-ol 8-*O*-β-d-gluco-pyranoside (**3**), and 7-[(β-d-apiofuranosyl-(1→6)-β-d-glucopyranosyl)oxymethy]-8,8-dimethyl-4,8-dihydrobenzo[1,4]thiazine-3,5-dione (**4**), were isolated from the fruits of *Xanthium sibiricum* Patr together with three known compounds, xanthiside (**5**), adenosine (**6**), and 2,3-dihydroxy-1-(4-hydroxy-3-methoxyphenyl)-propan-1-one (**7**). The structures of the new compounds were determined on the basis of detailed spectroscopic analyses.

## 1. Introduction

**Figure 1:**
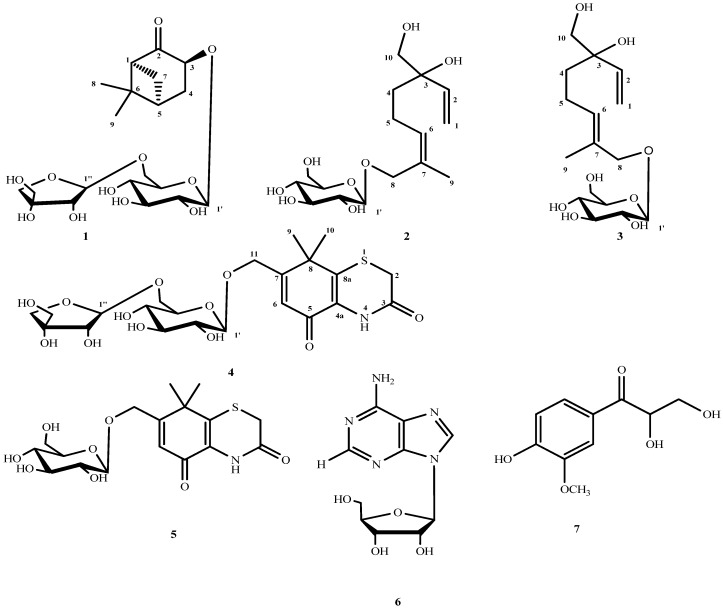
Structures of compounds **1**–**7** isolated from *Fructus Xanthii*.

The fruits of *Xanthium sibiricum* Patr. (Compositae), hereafter defined as “*Fructus Xanthii*”, known as *Cang er zi* in Traditional Chinese Medicine, are used for treating nasal sinusitis, numbness of limbs, arthritis, ulcer, pruritus, cancer, and herpes [1,2,3,4,5,6]. Many chemical studies on *Fructus Xanthii* have been conducted and several essential oils, amino acids, organic acids, sesquiterpene lactones, diterpenes, and thiazinediones have been isolated [7,8,9,10,11,12,13,14,15,16,17]. Moreover, the *n*-BuOH fraction of *Fructus Xanthii* has been shown to possess anti-inflammatory properties [18], and has been used for controlling macrophage-mediated inflammatory diseases [19]. However, there are few reports on the screening of bioactive components of *X. sibiricum*. Our study, which focused on the evaluation of anti-inflammatory properties of *Fructus Xanthii*, aimed to determine the most active constituents of the plant. Herein, we present the isolation and structure elucidation of four new compounds: 3β-norpinan-2-one 3-*O*-β-d-apiofuranosyl-(1→6)-β-d-glucopyranoside (**1**), (6*Z*)-3-hydroxymethyl-7-methylocta-1,6-dien-3-ol 8-*O*-β-d-glucopyranoside (**2**), (6*E*)-3-hydroxymethyl-7-methylocta-1,6-dien-3-ol 8-*O*-β-d-gluco-pyranoside (**3**), and 7-[(β-d-apiofuranosyl-(1→6)-β-d-glucopyranosyl)oxymethy]-8,8-dimethyl-4,8-dihydrobenzo[1,4]thiazine-3,5-dione (**4**), as well as three known compounds: xanthiside (**5**) [14], adenosine (**6**) [20], and 2,3-dihydroxy-1-(4-hydroxy-3-methoxyphenyl)-propan-1-one (**7**) [21], from the ethanol extract of *Fructus Xanthii* (Figure 1).

## 2. Results and Discussion

**Figure 2:**
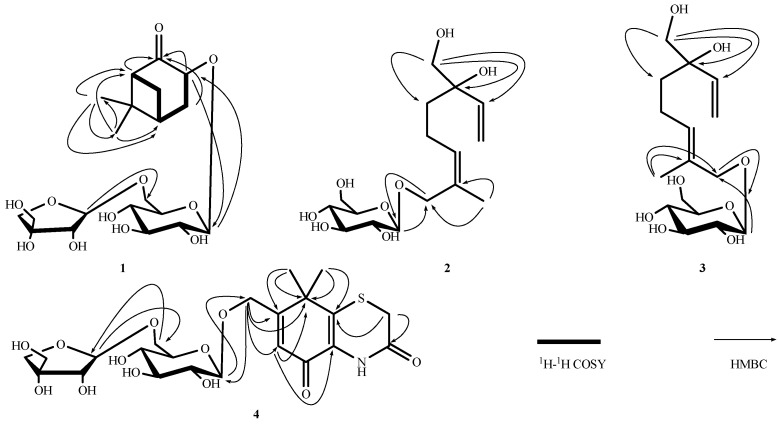
Key ^1^H-^1^H COSY and HMBC correlations of compounds **1**–**4**.

**Figure 3:**
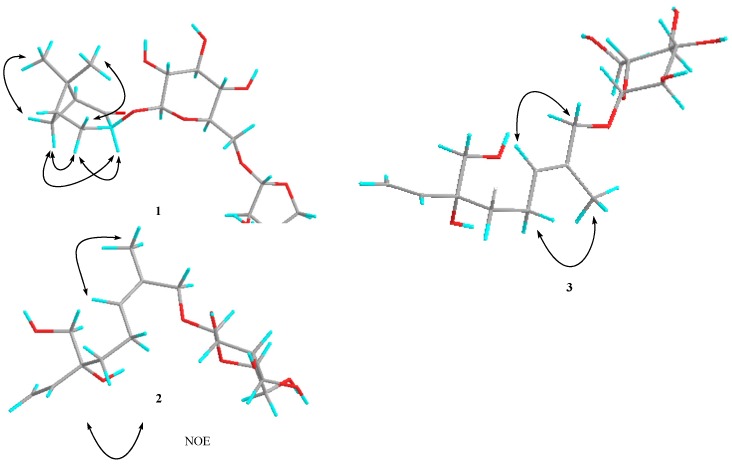
Main NOE correlations of compounds **1**–**3**.

Compound **1** was obtained as a white amorphous powder with molecular formula C_20_H_32_O_11_ (deduced from the HRESIMS and NMR data). The ^1^H-NMR spectrum showed proton signals for two methyl groups at δ 0.79 (3H, s, Me-8) and 1.36 (3H, s, Me-9), and two anomeric proton signals of sugar moieties at δ 4.50 (1H, d, *J* = 7.8 Hz, H-1') and 5.01 (1H, d, *J* = 2.5 Hz, H-1'). The ^13^C-NMR spectrum of **1** displays 20 carbon signals separated by DEPT experiments into two methyl, five methylene, 10 methine, and three quaternary carbon moieties. The structure of **1** was also confirmed by 2D NMR experiments (Figure 2). In the ^1^H-^1^H COSY spectrum of **1**, correlations were observed from H-1 to H-7, H-7 to H-5, H-5 to H_2_-4, and H_2_-4 to H-3. In the HMBC spectrum, some key correlations were observed from H-1 to C-2, C-3, C-5, C-6 and C-7; H-3 to C-2 and C-1'; H_2_-4 to C-2, C-3, C-5, and C-6; H-5 to C-1; H_2_-7 to C-1, C-2, C-4, and C-5; H_3_-8 to C-1, C-5, and C-9; H_3_-9 to C-1, C-5, and C-8; H-1' to C-3; and H-1' to C-6'. From the analysis of the ^1^H-^1^H COSY and HMBC spectral data, the planar structure of compound **1** was determined. Therefore, the aglycone of **1** was concluded to be 3-hydroxynorpinan-2-one [22], and the location of its glycosyl group was found to be C-3. The NOESY correlations between H_3_-8 and H_β_-4, H_3_-9 and H_β_-7, H_α_-4 and H_α_-7, H_α_-4 and H-3, and between H_α_-7 and H-3 established the connective sites, as shown in the structure of **1** (Figure 3). Therefore, the relative configuration of H-3 is α. Acid hydrolysis of **1** liberated d-glucose and d-apiose, which were identified by HPLC with optical rotation detection [23,24]. By comparing the coupling constants and chemical shifts of sugar signals with those of the reported sugars [25,26], the two sugars were deduced to be the β-configuration of glucose and β for apiose. From these results, **1** was determined to be 3β-norpinan-2-one 3-*O*-β-d-apiofuranosyl-(1→6)-β-d-glucopyranoside.

Compound **2** was obtained as a yellow amorphous powder with molecular formula C_16_H_28_O_8_ (determined by the HRESIMS and NMR data). Acid hydrolysis of **2** liberated d-glucose, which was identified by HPLC with optical rotation detection [24]. The ^1^H-NMR spectrum indicates the presence of a vinyl group, δ 5.15 (1H, dd, *J* = 1.7, 10.9 Hz, H-1a), 5.27 (1H, dd, *J* = 1.7, 17.4 Hz, H-1b), and 5.89 (1H, dd, *J* = 10.9, 17.4 Hz, H-2); an additional olefinic hydrogen, δ 5.41 (1H, brt, *J* = 7.0 Hz, H-6); four methylene protons, δ 1.54 (1H, ddd, *J* = 9.6, 6.0, 2.4 Hz, H-4a), 1.57 (1H, ddd, *J* = 9.6, 6.0, 2.4 Hz, H-4b), 2.05 (1H, m, H-5a), and 2.15 (1H, m, H-5b); two oxygenated methylene groups, δ 3.40 (2H, d, *J* = 2.6 Hz, H_2_-10), 4.35 (1H, d, *J* = 11.4 Hz, H-8a ), and 4.20 (1H, d, *J* = 11.4 Hz, H-8b ); one methyl group, δ 1.76 (3H, s, H-9); and an anomeric proton of a gluco-pyranoside, δ 4.21(1H, d, *J* = 7.8 Hz H-1'). From the coupling constant of the anomeric proton, C-1' of d-glucose was determined to be in the β-configuration. In the ^13^C-NMR and DEPT spectra, 16 signals except six signals resulting from the glucopyranosyl moiety were observed. These peaks suggest the presence of an aliphatic monoterpene skeleton. Thus, signals for two sets of double bonds [C-1 (δ 114.5), C-2 (δ 142.6), C-6 (δ 131.5), C-7 (δ 132.7)], two methylene [C-4 (δ 38.2), C-5 (δ 22.8)], two oxygenated methylenes [C-8 (δ 67.8), C-10 (δ 69.5)], one methyl [C-9 (δ 21.9)] moieties were observed, together with a quaternary carbon signal at δ 76.7 (C-3). In the HMBC spectrum, the key correlations were observed from H_3_-9 to C-7 and C-8; H_2_-10 to C-2, C-3, and C-4; and H-1' to C-8. Therefore, the glycosyl group is located at C-8. A diagnostic cross peak was observed between the methyl hydrogen (H_3_-9) and the olefinic hydrogen (H-6) in the NOESY spectrum (Figure 3). Thus, the stereochemistry of the double-bond system between C-6 and C-7 in **2** was confirmed to have the *Z* orientation. Even though the configuration of C-3 of **2** was not defined, this is the first report of the isolation of an acyclic monoterpene glycoside from *Fructus Xanthii*. Therefore, compound **2** was determined to be (6*Z*)-3-hydroxymethyl-7-methylocta-1,6-dien-3-ol 8-*O*-β-d-glucopyranoside.

Compound **3** was also obtained as a yellow amorphous powder. The positive-ion HRESIMS of **3** exhibits a quasi-molecular ion peak at *m*/*z* 371.1685 [M+Na]^+^. The molecular formula of **3** (C_16_H_28_O_8_) was determined from the quasi-molecular ion peak and HRESIMS measurements. Acid hydrolysis of **3** liberated d-glucose, which was identified by HPLC with optical rotation detection [24]. ^1^H-NMR (CD_3_OD) and ^13^C-NMR spectra (Table 1 and Table 2) of **3**, which were examined using the results of various NMR experiments, show signals assignable to an aglycone moiety [δ 5.30 (1H, dd, *J* = 1.6, 17.4 Hz, H-1a), 5.17 (1H, dd, *J* = 1.6, 10.9 Hz, H-1b)], [δ 5.88 (1H, dd, *J* = 17.4, 10.9 Hz, H-2)], [δ 1.59 (1H, ddd, *J* = 9.6, 6.0, 2.4 Hz, H-4a), 1.52 (1H, ddd, *J* = 9.6, 6.0, 2.4 Hz, H-4b)], [δ 2.06 (1H, m, H-5a), 2.16 (1H, m, H-5b)], [δ 5.48 (1H, brt, *J* = 6.5 Hz, H-6)], [δ 4.18 (1H, d, *J* = 11.5 Hz, H-8a), 4.03 (1H, d, *J* = 11.5 Hz, H-8b)], [δ 1.68 (3H, s), H-9], [δ 3.41 (2H, d, *J* = 3.2 Hz)] and a β-d-glucopyranosyl moiety [δ 4.23 (1H, d, *J* = 7.8 Hz)]. All ^1^H-NMR and ^13^C-NMR data were similar to those for 2. However, an important difference observed for this part was the chemical shift value of the vinyl methyl carbon, δ 14.1 (C-9). Analysis of the ^13^C-NMR spectra revealed signal values similar to those reported for creoside II [27]; thus, the double bond of **3** was determined to be *trans*. In addition, the geometry of **3** was also confirmed on the basis of NOESY results, which showed NOE correlations between the following proton pairs: H_2_-5 and H_3_-9; H-6 and H_2_-8 (Figure 3). Thus, the stereochemistry of the double bond system between C-6 and C-7 in **3** was confirmed to be of the *E* form. Thus, compound **3** was determined to be (6*E*)-3-hydroxymethyl-7-methylocta-1,6-dien-3-ol 8-*O*-β-d-glucopyranoside.

**Table 1:** ^1^H-NMR data for compounds **1**–**4** (400 MHz in CD_3_OD).

No.	1	2	3	4
1	2.58 (1H, t, *J* = 5.4 Hz)	5.27 (1H, dd, *J* = 1.7, 17.4 Hz)	5.30 (1H, dd, *J* = 1.6, 17.4 Hz)	
		5.15 (1H, dd, *J* = 1.7, 10.9 Hz)	5.17 (1H, dd, *J* = 1.6, 10.9 Hz)	
2		5.89 (1H, dd, *J* = 17.4, 10.9 Hz)	5.88 (1H, dd, *J* = 17.4, 10.9 Hz)	3.48 (2H, brs)
3	4.70 (1H, dd, *J* = 6.9, 10.6 Hz)			
4	1.90 (1H, dd, *J* = 6.9, 13.6 Hz)	1.57 (1H, ddd, *J* = 9.6, 6.0, 2.4 Hz)	1.59 (1H, ddd, *J* = 9.6, 6.0, 2.4 Hz)	
	2.77 (1H, ddd, *J* = 4.6, 10.6, 13.6 Hz)	1.54 (1H, ddd, *J* = 9.6, 6.0, 2.4 Hz)	1.52 (1H, ddd, *J* = 9.6, 6.0, 2.4 Hz)	
5	2.22 (1H, m)	2.05 (1H, m)	2.06 (1H, m)	
		2.15 (1H, m)	2.16 (1H, m)	
6		5.41 (1H, brt, *J* = 7.0 Hz)	5.48 (1H, brt, *J* = 6.5 Hz)	6.67 (1H, brs)
7	2.48 (1H, m)			
	1.81 (1H, m)			
8	0.79 (3H, s)	4.35 (1H, d, *J* = 11.4 Hz)	4.18 (1H, d, *J* = 11.5 Hz)	
		4.20 (1H, d, *J* = 11.4 Hz)	4.03 (1H, d, *J* = 11.5 Hz)	
9	1.36 (3H, s)	1.76 (3H, s)	1.68 (3H, s)	1.47 (1H, s)
10		3.40 (2H, d, *J* = 2.6 Hz)	3.41 (2H, d, *J* = 3.2 Hz)	1.47 (1H, s)
11				4.70 (1H, d, *J* = 15.8 Hz)
				4.51 (1H, d, *J* = 15.8 Hz)
Glc-1'	4.50 (1H, d, *J* = 7.8 Hz)	4.21 (1H, d, *J* = 7.8 Hz)	4.23 (1H, d, *J* = 7.8 Hz)	4.35 (1H, d, *J* = 7.7 Hz)
2'	3.24 (1H, m)	3.15 (1H, dd, *J* = 8.9, 7.8 Hz)	3.18 (1H, m)	3.30 (1H, m)
3'	3.35 (1H, m)	3.25 (1H, m)	3.25 (1H, m)	3.34 (1H, m)
4'	3.28 (1H, m)	3.30 (1H, m)	3.31 (1H, m)	3.25 (1H, m)
5'	3.40 (1H, m)	3.35 (1H, m)	3.34 (1H, m)	3.41 (1H, m)
6'	3.98 (1H, dd, *J* = 12.5, 2.0 Hz)	3.87 (1H, dd, *J* = 12.5, 2.0 Hz)	3.85 (1H, dd, *J* = 12.0, 2.2 Hz)	3.98 (1H, dd, *J* = 12.9, 1.6 Hz)
	3.60 (1H, dd, *J* = 12.5, 5.0 Hz)	3.67 (1H, dd, *J* = 12.5, 5.0 Hz)	3.65 (1H, dd, *J* = 12.0, 5.6 Hz)	3.61 (1H, dd, *J* = 11.4, 6.5 Hz)
Api-1''	5.01 (1H, d, *J* = 2.5 Hz)			5.01 (1H, d, *J* = 2.4 Hz)
2''	3.89 (1H, d, *J* = 2.5 Hz)			3.89 (1H, d, *J* = 2.4 Hz)
3''				
4''	3.74 (1H, d, *J* = 9.6 Hz)			3.95 (1H, d, *J* = 9.6 Hz)
	3.97 (1H, d, *J* = 9.6 Hz)			3.74 (1H, d, *J* = 9.6 Hz)
5''	3.56 (2H, s)			3.48 (2H, s)

**Table 2:** ^13^C-NMR data for compounds **1**–**4** (100 MHz in CD_3_OD).

No.	1	2	3	4
1	57.9	114.5	114.5	
2	215.2	142.6	142.5	29.8
3	78.5	76.7	76.7	164.7
4	32.9	38.2	37.5	
4a				131.0
5	41.4	22.8	22.8	177.1
6	45.5	131.5	130.3	123.4
7	24.9	132.7	132.9	167.2
8	21.9	67.8	75.9	43.5
8a				143.5
9	26.3	21.9	14.1	27.5
10		69.5	69.7	27.4
11				67.9
Glc-1'	105.9	102.4	102.6	103.9
2'	75.5	75.0	75.1	75.1
3'	77.9	77.9	77.9	78.0
4'	71.6	71.7	71.7	71.7
5'	77.1	78.2	78.2	77.2
6'	68.7	62.8	62.8	68.7
Api-1''	111.0			111.0
2''	78.0			78.0
3''	80.5			80.5
4''	75.0			75.0
5''	65.5			65.5

Compound **4** was obtained as colorless needle-like crystals with molecular formula C_22_H_31_NO_12_S (deduced from the HRESIMS and NMR data). The ^1^H-NMR spectrum of **4** reveals signals for an olefinic proton, δ 6.67 (1H, brs, H-6); two methyls of a *gem*-dimethyl group, δ 1.47 (6H, s, H_3_-9, 10); two oxygenated methylene protons, δ 4.70/4.51 (each 1H, d, *J* = 15.8 Hz, H-11a/H-11b) and δ 3.48 (2H, brs, H-2); and two anomeric proton signals of sugar moieties at δ 4.35 (1H, d, *J* = 7.7 Hz, H-1') and 5.01 (1H, d, *J* = 2.4 Hz, H-1'). The ^13^C-NMR spectrum displays 22 carbon signals. Based on the DEPT and HSQC analyses, these signals were assigned to two double bonds at δ 123.4 (C-6), 167.2 (C-7), 131.0 (C-4a), and 143.5 (C-8a); an oxygenated methylene, δ 67.9 (C-11); an aliphatic methylene, δ 29.8 (C-2); two methyls of a *gem*-dimethyl group, δ 27.5 (C-9) and 27.4 (C-10); an aliphatic quaternary carbon, δ 43.5 (C-8); and two quaternary carbons at δ 164.7 (C-3) and 177.1 (C-5). Comparison of these data with those of thiazinedione, which was previously isolated from *Xanthium strumarium* [5], indicated that we are dealing with a glycoside derivative of thiazinedione. In the HMBC spectrum (Figure 2), the key correlations were observed from H_3_-9/10 to C-8, C-7, and C-8a; H_2_-11 to C-8, C-7, and C-6; H-6 to C-8, C-4a, and C-11; and H_2_-2 to C-8a and C-3. One of the important correlations of HMBC was observed from the anomeric proton (H-1' to C-11), and the other correlation was observed from the anomeric proton (H-1') to the position of glucose (C-6'). Indeed, acid hydrolysis of **4** afforded glucose and apiose. The specific rotations of glucose and apiose revealed that they were d-sugars [23,24]. The glycosyl groups were deduced from the ^13^C-NMR spectroscopic data and anomeric proton coupling constants as glucose and apiose having β-anomeric configuration [25,26]. Therefore, the new compound **4** was identified as 7-[(β-d-apiofuranosyl-(1→6)-β-d-glucopyranosyl)oxymethy]-8,8-dimethyl-4,8-dihydrobenzo[1,4]thiazine-3,5-dione.

## 3. Experimental

### 3.1. General

IR spectra were recorded on an IR-47 spectrometer. An optical-rotation detector (Shodex OR-2, Showa Denko Co., Ltd., Tokyo, Japan) was used. The optical rotation was recorded on a Perkin-Elmer 241 polarimeter. The melting points (uncorrected) were measured on a Kofler micromelting point apparatus. The HRESIMS analyses were conducted on an IonSpec Ultima 7.0T FTICR. The UV and NMR spectra were recorded on a Shimadzu UV-1601 and Bruker DPX 400 (400 MHz for ^1^H-NMR and 100 MHz for ^13^C-NMR), respectively. Chemical shifts are given as δ values, with reference to that of tetramethylsilane (TMS), which was used as an internal standard. Coupling constants are given in Hz. Preparative HPLC (Waters, Delta 600-2487) was performed on a Hypersil-ODS II column (10 μm, 20 × 300 mm, Yilite, Dalian, China). Semipreparative HPLC (Waters, Delta 600-2414-2998) was performed using a SunFire^TM^-C18 column (5 μm, 10 × 250 mm, Waters, Milford, MA., USA). Silica gel (200–300 mesh, Yanghai, Qingdao, China) and ODS-A (120 A, 50 µm, YMC Co., Tokyo, Japan) were employed for column chromatography (CC).

### 3.2. Plant Material

*Fructus Xanthii* was collected from Heilongjiang Province, China, in August 2011, and then authenticated by Prof. Wang Zhen-Yue, Heilongjiang University of Chinese Medicine, Harbin, China. A voucher specimen (No. 20111077) was deposited at the herbarium of Heilongjiang University of Chinese Medicine.

### 3.3. Extraction and Isolation

A 70% aqueous EtOH extract of *Fructus Xanthii* (2.7 kg) was partitioned between EtOAc and H_2_O and between *n*-BuOH and H_2_O. The *n*-BuOH extract (59.4 g) was subjected to silica gel chromatography using CH_2_Cl_2_–MeOH mixtures [20:1 (10 L), 10:1 (7 L), 5:1 (15 L), 2:1 (5 L) v/v] to afford fractions (Fr.) A–D. Fr.A was stored and Fr.B (12.1 g) was repeatedly subjected to CC on an ODS column using MeOH–H_2_O solution [1:7 (3 L)–5:6 (4 L) v/v] as the mobile phase to afford compounds **1** (9 mg) and **6** (24 mg). Fr.C (20.3 g) was subjected to silica gel chromatography using CH_2_Cl_2_–MeOH mixtures [15:1 (1.8 L), 5:1 (2.8 L) v/v] to afford subfractions C1 (8.2 g) and C2 (9.7 g). Fr.C2 was repeatedly subjected to separation using Sephadex LH-20 and CH_2_Cl_2_–MeOH solution [1:1 (0.7 L) v/v] to yield Fr.C2-1 (4.6 g) and Fr.C2-2 (3.7 g). Fr.C2-2 was subjected to isocratic elution on an ODS column using MeOH–H_2_O solution [2:1 (0.5 L) v/v] to afford Fr.C2-2-1 (1.0 g). Fr.C2-2-1 was finally purified by preparative HPLC using MeOH–H_2_O solution (3:7, 6 mL/min, *t*_R_ = 8.5 min; *t*_R_ = 9.5 min) to afford compounds **2** (3.2 mg) and **3** (4.1 mg). Fr.D (10.7 g) was loaded on a Sephadex LH-20 column and eluted with CH_2_Cl_2_–MeOH solution [1:1 (0.8 L)] to yield five fractions, D1–D5. D2 (300 mg) was purified by semipreparative HPLC (isocratic elution) using MeOH–H_2_O solution (2:3, 3 mL/min, t_R_ = 12.7, 18.0, and 15.0 min) to afford compounds **4** (10.0 mg), 7 (19.0 mg), and **5** (57.1 mg).

*3β-Norpinan-2-one 3-O-β-d-apiofuranosyl-(1→6)-β-d-glucopyranoside* (**1**): white amorphous powder 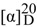 −10.2 (c 0.26, MeOH) UV λ_max_ (MeOH) nm (log ε): 201(3.18) IR (KBr): 3560, 3495, 2980, 1713, 1075, 1042 cm^−1^ ^1^H-NMR: Table 1 ^13^C-NMR: Table 2 HRESIMS *m*/*z* [M+Na]^+^ Calcd for C_20_H_33_O_11_Na 471.1842, Found: *m*/*z* 471.1847.

*(6Z)-3-Hydroxymethyl-7-methylocta-1,6-dien-3-ol 8-O-β-d-glucopyranoside* (**2**): yellow amorphous powder 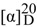 −6.7 (c 0.17, MeOH) UV λ_max_ (MeOH) nm (log ε): 202(2.10) IR (KBr): 3378, 2980, 2830, 1050, 917; cm^−1^ ^1^H-NMR: Table 1 ^13^C-NMR: Table 2 HRESIMS *m*/*z* [M+Na]^+^ Calcd for C_16_H_28_O_8_Na 371.1682, Found: *m/z* 371.1680.

*(6E)-3-Hydroxymethyl-7-methylocta-1,6-dien-3-ol 8-O-β-d-glucopyranoside* (**3**): yellow amorphous powder 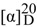 −8.0 (c 0.19, MeOH) UV λ_max_ (MeOH) nm (log ε): 201(2.10) IR (KBr): 3380, 2979, 2833, 1056, 919; cm^−1^ ^1^H-NMR: Table 1 ^13^C-NMR: Table 2 HRESIMS *m*/*z* [M+Na]^+^ Calcd for C_16_H_28_O_8_Na 371.1682, Found: *m*/*z* 371.1685.

*7-[(β-d-Apiofuranosyl-(1→6)-β-d-glucopyranosyl)oxymethy]-8,8-dimethyl-4,8-dihydrobenzo[1,4]thiazine-3,5-dione* (**4**): colorless needle crystals mp 190–192 °C 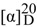 −10.7 (c 0.22, MeOH) UV λ_max_ (MeOH) nm (log ε): 201(2.18), 246(2.87) IR (KBr): 3462, 1690; 1658, 1215, 1177, 929 cm^−1^ ^1^H-NMR: Table 1 ^13^C-NMR: Table 2 HRESIMS *m*/*z* [M+H]^+^ Calcd for C_22_H_32_NO_12_S 534.1645, Found: *m*/*z* 534.1650.

### 3.4. Acid hydrolysis of ***1**, **2**, **3**,* and ***4***

Compound **1** (3.0 mg) was dissolved in 2 M HCl (2.0 mL) and then heated at 80 °C in a water bath for 2 h. The reaction mixture was diluted with H_2_O (20 mL) and then extracted with *n*-BuOH (20 mL). The aqueous layer was neutralized with Amberlite MB-3 (Organo Co., Ltd., Tokyo, Japan), and then dried under reduced pressure to afford the monosaccharide fraction. The monosaccharide fraction was extracted with MeOH, and the MeOH extract was analyzed using HPLC under the following conditions: HPLC column, Kaseisorb LC NH_2_-60-5 (4.6 mm i.d. 250 mm; Tokyo Kasei Co., Ltd., Tokyo, Japan); detection, optical rotation (Shodex OR-2, Showa Denko Co., Ltd., Tokyo, Japan); mobile phase, CH_3_CN–H_2_O (17:3, v/v); flow rate, 1.0 mL/min. Identification of d-glucose and d-apiose from **1** was carried out by comparing their retention times and optical rotations with those of authentic samples. The values of *t*_R_ were 11.5 min (d-glucose, positive optical rotation) and 5.8 min (d-apiose, positive optical rotation). Through the same method, the monosaccharides were identified as d-glucose for **2** and **3**, and d-glucose and d-apiose for **4**.

## 4. Conclusions

As a part of our chemical investigation on *Fructus Xanthii*, four new glycosides, namely 3β-norpinan-2-one 3-*O*-β-d-apiofuranosyl-(1→6)-β-d-glucopyranoside (**1**), (6*Z*)-3-hydroxymethyl-7-methylocta-1,6-dien-3-ol 8-*O*-β-d-glucopyranoside (**2**), (6*E*)-3-hydroxymethyl-7-methylocta-1,6-dien-3-ol 8-*O*-β-d-glucopyranoside (**3**), and 7-[(β-d-apiofuranosyl-(1→6)-β-d-glucopyranosyl)oxymethy]-8,8-dimethyl-4,8-dihydrobenzo[1,4]thiazine-3,5-dione (**4**) were isolated. Their structures were established on the basis of spectroscopic evidence. This discovery represents a further addition to the number and diversity of glycosidic compounds.
